# Welcome to the special issue on organs-on-chip from the guest editors

**DOI:** 10.1016/j.stemcr.2021.08.013

**Published:** 2021-09-14

**Authors:** Christine Mummery, Peter Loskill

**Affiliations:** President ISSCR 2020–2021, Chair EUROoCS 2018–2021; Chair EUROoCS 2021–2024

## Main text

### Go with the flow: Human tissue models beyond cells alone

Stem cell models to understand development and disease are now central to many studies investigating underlying molecular mechanisms of these processes and screening drugs or identifying drug targets that modify the normal trajectory cells take during their lifespan. But many models are based on culturing stem cells or their differentiated derivatives on tissue culture plastic substrates in two dimensional (2D) monolayers. Recent studies with organoids from both human induced pluripotent stem cells (hiPSCs) and adult stem cells have demonstrated how much more realistic tissue mimics can be in 3D, especially when they are recapitulating spatial organization of the cell types and the extra cellular matrix (ECM) normally present in the tissue as well as the mechanical properties of the cellular microenvironment. Groundbreaking discoveries have already emerged from studying organoids and as a result, there is a far better understanding of cell interactions that take place in human organ development, how self-organization takes place, and which cell types are disease “culprits” versus “victims.” Organoids, however, miss (micro)fluidic flow that mimics the flow of fluid or blood through the tissue. In addition, they miss physiological compartmentalization and biomechanical stimulation from stretch and contraction that occurs in tissues such as the heart or lungs. That is what organs-on-chip (OoC) models are designed to do: incorporate (micro) channels for fluid or air flow, provide *in-vivo*-like compartmentalization, and provide the biomechanical environment of real organs. In fact, one of the first OoCs was a lung-on-chip model: lung epithelial cells were grown on a flexible membrane, with fluid flow on one side, airflow on the other, and two side channels with an oscillating vacuum that mimicked the breathing movement of a lung. In an interview (2047), Don Ingber, inventor of the lung-on-chip model and founder of the Wyss Institute for Biologically Inspired Engineering at Harvard, describes how he first realized that mechanical “instructions” were incredibly important in biology, regulating how living cells organize into tissues and how embryos form. In another interview (2044), Matthias Lutolf, a professor of bioengineering at EPFL in Lausanne and recently appointed as the scientific director of the Roche Institute for Translational Bioengineering, describes his step across disciplinary boundaries and sectors. This led to many high-profile studies from his lab in which carefully engineered structures of biomaterials were combined with stem cells, resulting in amazingly realistic models of the human intestine and even embryos (see also the May 11, 2021 special issue of *Stem Cell Reports* on embryo models).

### Organs-on-chip

**Figure 1:**
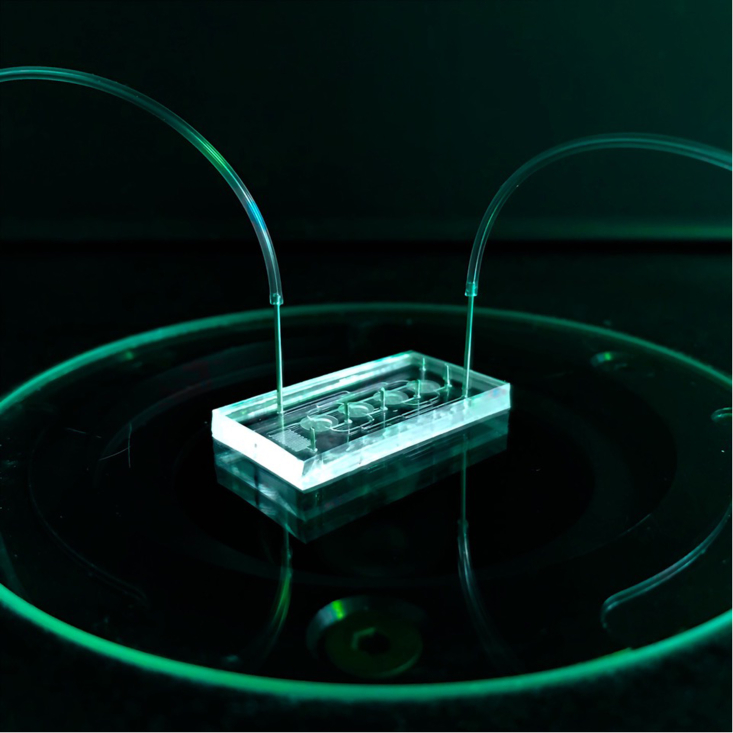
An OoC device with microchannels, together with the attached tubes that are the inflow and outflow outlets for fluids The chambers containing cells are visible in the middle and the microchannels may be lined with endothelial cells. The fluid can be blood or culture medium, with or without, for example, immune cells, drugs, viruses, or bacteria.

OoCs and organoids are subcategories of microphysiological systems (MPSs). In contrast to organoids, OoCs are microfluidic platforms featuring narrow channels through which fluid can flow and compartments for 2D or 3D cell culture to mimic tissue and organ level physiology. They have conventionally been fabricated using photolithography and replica molding of a silicon rubber called Polydimethylsiloxane (PDMS) (see Figure 1). More recently, other materials and methods have been used. OoCs have been used to study cell-biomaterial interactions and tissue properties such as perfusion but have also been designed to generate chemotactic gradients, fluid sheer stress, and localized mechanical and electrical stimulation. More sophisticated variants now also contain nanosensors to detect changes in cell behavior non-invasively.

### Stem cells in organ-on-chip

Most of the early OoC models incorporated primary (human) cells or cell lines and this yielded a mass of new information on how biophysical properties of human tissues affected physiological and pathophysiological processes. It also allowed screening of drugs and looking at the effects of bacteria, viruses, and immune cells under conditions of microfluidic flow. But sourcing primary human tissues is not always easy. Proper informed consent is required, which is not always straightforward for companies. How large cell stocks are depends on how well the cells grow in culture without changing properties. And for some tissues, like brain or eye, obtaining tissue biopsies is challenging. Immortalized or cancer cell lines are alternatives but these are often very different from normal cells in the body.

With the advent of straightforward and robust protocols and methods for obtaining differentiated cells from adult or pluripotent stem cells, the OoC landscape has started to change. Stem cells are no longer just the domain of biologists but have become accessible to bioengineers, biotech companies, and pharma. They are available commercially, sometimes with genetic reporters of differentiation state or subcellular structures. Kits to induce their differentiation are sold by several suppliers and even cryopreserved cells, ready to go for implementation in assays simply after thaw, are readily available. The time is right to merge these two fields: OoCs filled with specific types of cells under near physiological conditions can now be produced from any genetic background from which stem cells are available. This not only includes genotypes bearing specific disease mutations but also those with specific disease predispositions. We have learned that perhaps there is no such thing in stem cell research as a “healthy control” but, as we have learned more about the genome, there may be genotypes with large numbers of variants (or “SNPs”) predisposing for a disease and others with none. That would then be defined as the healthy control for that individual and OoC models with those cells may be able to tell us something about the triggers that lead to disease onset, beyond that of standard models.

In this special issue, a number of excellent examples are featured that showcase the potential of MPSs that merge stem cell and microfabrication technologies:

• Engineered cardiac tissues, in which the heart phenotype of Duchenne’s Muscular Dystrophy (DMD) is revealed if DMD hiPSC-derived cardiomyocytes are aligned with the correct aspect ratio of 1:7 on a substrate with the same stiffness as the heart in DMD patients, are possible. (See Chang et al., 2169).
• Three-dimensional hiPSC-derived neuronal networks (combined with micro-electrode arrays) allow the study of disease-specific genotype-phenotype correlations or the effect of treatments (see Mossink et al., 2182, and Gunneweik et al., 2197).
• Complex models of the retina and its retinofugal projections enable the assessment of genetic conditions of the eye and suitable gene therapies (see Fligor et al., 2228, and Achberger et al., 2242).
• Parallelization of organoid handling and analysis allows high-throughput screening on, for example, cochlear organoids (see Q. Liu et al., 2257).
• Integration of disease-specific stem cells into microfluidic modules allows the effect of individual gene mutations to be studied, e.g. on human motor units (see Stoklund Dittlau et al., 2213).

In every case, the fluid channels in the microfluidic modules benefit from being lined with vascular cells specific for the tissue of interest (see Browne et al., 2058). While it has long been realized that vascular endothelial cells differ between arteries, veins, and capillaries, the more subtle differences between vessels in different organs and tissue types is now only just emerging with the increasing availability of single-cell sequencing data from tissue biopsies. hiPSCs can form endothelial cells and the surrounding vascular smooth muscle cells that serve to stabilize the vessel very efficiently: by recapitulating developmental signals, not only can we make specialist vascular cells of heart and kidney, but also of brain. Vila Cuenca et al. (2159) show how elegantly vascular endothelial cells efficiently self-organize into 3D vessels with lumen in a commercially available OoC system, but these vessels will interact with hiPSC-derived vascular smooth muscle in a way that is indistinguishable from interaction with primary human brain vascular smooth muscle. It is thus becoming possible to create complex MPS models including OoCs that are entirely isogenic, i.e. derived from the same hiPSC source and genetic background.

### Impact and future of OoCs

While for many years the work with OoCs required specialized engineering laboratories with clean room facilities to manufacture the microfluidic modules, an increasing number of OoCs are now available commercially, requiring sometimes solely standard cell culture facilities and sometimes more complex pump infrastructure to exert pressure and drive fluid through the channels. These systems are now poised for increased utility that will emerge as they are standardized and parallelized further and become amenable to higher-throughput experimentation (see Piergiovanni et al., 2076 and Mastrangeli et al., 2037). That the concept of merging stem cells, organoids, and OoCs is not just “academic reverie” but truly relevant much more widely is illustrated by the increasing adoption of the approach by industry. This is particularly evident for the heart where models containing hiPSC-derived cardiomyocytes are close to regulatory acceptance as alternatives to mice for cardiotoxicity prediction (see Stein et al., 2049). The inclusion of fluid channels containing immune cells flowing through the system and forcing the beat rate to increase from the standard 60 beats per minute to, say, 180 will undoubtedly increase the value of these models even further. By becoming a more mature technology, OoCs now frequently transition from developer laboratories into those of end-users from academia or pharmaceutical industry where they have a direct impact on patient health as well as the 3Rs (Reduce, Refine, Replace animal experiments). Especially for the latter, the combination of stem cells, organoids, and organ-on-chip provides the opportunity for potentially radical changes in the way novel drug are discovered and developed (see Loskill et al., 2033).

### European Organ on Chip Society: EUROoCS

EUROoCS was founded in 2018 to unite many of the diverse national and European OoC initiatives. Just like the ISSCR, but on a smaller scale, EUROoCS organizes an annual conference at which a few invited keynote speakers present but which mostly highlights the work of Ph.D. students, postdocs, and junior researchers who are the backbone of the program. EUROoCS also has an Industrial Advisory Board (IAB) that provides guidance on applications of OoC technology in industry. Thomas Singer (senior drug development expert, formerly of Roche) is founding chair of the IAB. Thomas strongly believes these models can support the 3R endeavors worldwide. Maurice Whelan heads the Regulatory Advisory Board (RAB) that creates a forum for EUROoCS to gather information on what would be necessary for acceptance of MPS models as part of the process of drug approval.

In 2020, ISSCR and EUROoCS reached an agreement to collaborate in combining publication of stem cell studies in OoC or MPS formats. Ten OoC researchers with stem cell backgrounds joined the editorial board of *Stem Cell Reports* to organize the review process for manuscripts. Members of EUROoCS are offered the opportunity for member discounts in their submissions to *Stem Cell Reports* and are delighted with the standard open access formula. The special issue here is the first product of this new initiative. We hope you enjoy browsing through the articles and are even inspired to implement some of the ideas in your own approach to creating *in vitro* models—and perhaps submitting the results of those studies to the regular issues of *Stem Cell Reports*.

**Figure 2:**
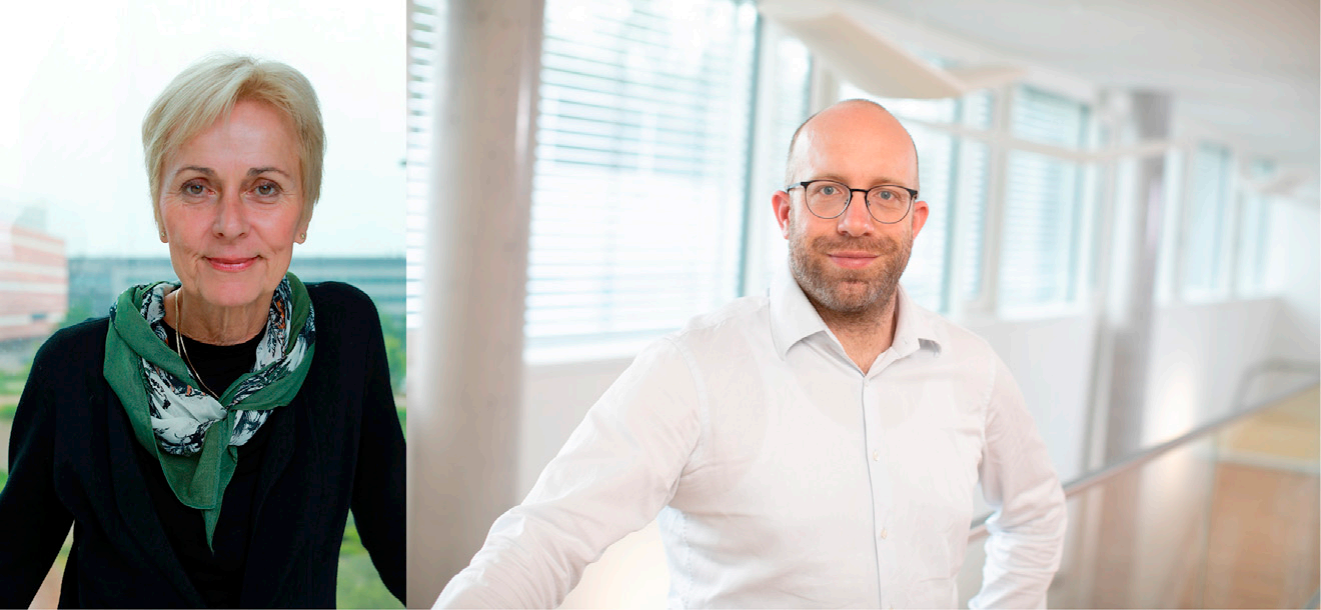
Christine Mummery (left) and Peter Loskill (right), guest editors of this special issue of *Stem Cell Reports*

As guest editors of this special issue of *Stem Cell Reports* (Figure 2), we are delighted to share with you a collection of papers and reviews on organs-on-chip or microphysiological systems that include organoids. This special issue marks the new collaboration between ISSCR and EUROoCS.

